# Impact of Green Tea Consumption on the Prevalence of Cardiovascular Outcomes: A Systematic Review

**DOI:** 10.7759/cureus.49775

**Published:** 2023-12-01

**Authors:** Hadrian Hoang-Vu Tran, Mafaz Mansoor, Samia Rauf R Butt, Travis Satnarine, Pranuthi Ratna, Aditi Sarker, Adarsh Srinivas Ramesh, Carlos Munoz, Dawood Jamil, Lubna Mohammed

**Affiliations:** 1 Internal Medicine, California Institute of Behavioral Neurosciences & Psychology, Fairfield, USA; 2 General Practice, California Institute of Behavioral Neurosciences & Psychology, Fairfield, USA; 3 Pediatrics, California Institute of Behavioral Neurosciences & Psychology, Fairfield, USA; 4 Family Medicine, Kamineni Academy of Medical Sciences and Research Center (KAMSRC), Hyderabad, IND

**Keywords:** cardiovascular diseases, catechin, dyslipidemia, green tea, hypertension

## Abstract

Cardiovascular diseases (CVDs) represent a major global health concern, responsible for significant morbidity, mortality, and disability. To mitigate the impact of CVDs, individuals often seek preventive measures, and one such approach is the consumption of green tea. This study aims to provide a comprehensive and up-to-date assessment of the effects of green tea consumption on the prevalence of cardiovascular outcomes.

Following PRISMA guidelines, we conducted a systematic review using PubMed and Google Scholar databases to identify relevant studies. Our analysis revealed that the risk factors associated with CVDs can vary across different diseases, with hypertension being a common risk factor for CVD mortality and CVD. Notably, the consumption of green tea exhibited a positive effect on reducing the prevalence of cardiometabolic risks and hypercholesterolemia. Furthermore, green tea consumption was observed to have a beneficial impact on lowering both diastolic and systolic blood pressure.

In conclusion, the studies reviewed in this research suggest that the consumption of green tea has a significant and positive influence on cardiovascular health. These findings highlight the potential of green tea as a valuable component of a healthy lifestyle, offering a promising avenue for its use as a dietary supplement to reduce the risk of CVDs.

## Introduction and background

Cardiovascular diseases (CVDs) affect the heart and blood vessels, including coronary artery disease, heart failure, stroke, and cardiomyopathy. CVDs are the principal worldwide causes of morbidity, mortality, and disability, with approximately 17.3 million deaths yearly [1]. Depending on the condition, the pathophysiologic mechanisms involved in CVDs differ. Hypertension is one of the most common causes of cardiovascular events and is responsible for 7,000,000 deaths worldwide [2]. Elevated blood pressure (BP) causes nearly half of all ischemic heart disease cases and 60% of stroke cases. The risk of CVDs doubles for every 20/10 mm Hg in BP from as low as 115/75 mmHg [3]. According to recent studies, even a minor reduction in BP is clinically significant in lowering the risk of coronary heart disease and stroke [4].

Hyperlipidemia is another significant risk factor for CVD, which is caused by lipid metabolism abnormalities and leads to the build-up of atherosclerotic plaques [5]. Previous studies have shown that patients with hyperlipidemia are three times more likely to have a heart attack than those with normal lipid levels [6]. Although multiple synthetic lipid-lowering drugs are on the market, their long-term use may cause various side effects [7].

For the prevention of CVD, lifestyle modifications and dietary interventions are beneficial [8]. As a result, there is an immense scientific and public interest in this topic [9]. Green tea has been studied for its anti-inflammatory, antihypertensive, cholesterol-lowering, and vasculoprotective effects, which may impact cardiometabolic risk [10]. Green tea, made from the Camellia sinensis plant, is a popular beverage worldwide and has long been used as a medicine in Asian countries [11]. Black tea is the most popular beverage in North America and Europe, green tea is famous in Asia, and oolong tea is popular in Southeast China [12]. Tea is consumed daily by approximately 21% of Americans, and tea consumers have shown a 20-fold higher flavonoid consumption than non-consumers [13]. Green tea is made from mature leaves that have undergone minimum processing (only drying) [14]. The flavonoid-like polyphenol contents in green tea, such as catechins, are primarily responsible for its therapeutic effects [15]. The most prevalent green tea extracts that have been found to show beneficial effects on health are epigallocatechin gallate (EGCG), epicatechin gallate, epigallocatechin, and epicatechin. However, caffeine also contributes to some of the impact [16].

With an aging population and the continuous risk of CVD as the primary contributor to worldwide morbidity and mortality, this study aims to assess and provide comprehensive and updated evidence on the impact of green tea consumption on the prevalence of cardiovascular outcomes. We conducted a systematic review.

## Review

Methods and results****

PRISMA were used in designing and reporting the results for the systematic review [17]. PRISMA guidelines and principles were adhered to regarding the inclusion of studies, exclusion of studies, extraction, and data analysis. The PRISMA guidelines were also attached to the reliability of the study's findings during the discussion of results. As published in the Cochrane Handbook for Systematic Reviews and interventions, the PRISMA extension was used in this systematic review - Chapter 4 [17].

Research Strategy

The study used PubMed and Google Scholar as the databases researched through electronic means and keywords and medical subject headings (MeSH) terms that were important for the study. PubMed Central was used to analyze full-text papers for those that had provided abstracts alone in PubMed. Hence, as identifies all the potential and relevant studies. The articles had to expound on the impact of green tea consumption and the risk of CVDs. The keywords include green tea, catechin, and CVDs. The identified studies were used after the screening to avoid dismissing articles relevant to the study. The most relevant articles were used for the analysis.

Inclusion and Exclusion Criteria

The studies used were limited to using only randomized cohort studies, systematic reviews, and metanalyses published from 2017 to 2022. Among the studies chosen, we ensured the study included participants above 18. The study participants were not limited to any confinements, whether a CVD history or a healthy participant. An exception was, however, made for the elderly participants as their ages had a high prevalence of CVD events. Studies that were to be included had also to have been published or translated into the English language. The population, intervention, control, outcomes, and studies (PICOS) was used as a crucial component in the criteria for eligibility. All duplicates were excluded from selected articles.

Quality Appraisal

Critical Appraisal Skills Programme (CASP) Standard Checklist [18] was used in the assessment for the quality of the study. The quality assessment tool uses the first three questions to validate the study's design. The following section uses three questions to assess the methodological soundness of the studies. Three more questions question the validity of the results, and the final two appraise the applicability of the findings. Questions in this checklist are answered using three answers, “Yes,” “No,” and “Can't tell.” The responses shall be abbreviated as Y, N, and CT. Using the 11-question checklist, the 10 studies were appraised for their quality of results and scored out of 11 [1,2,6,7,10,15,19-22]. A score of 11 indicated very high quality; 8 to 10 was considered high quality, 6 to 7 was moderate, and anything below 5 was regarded as low quality. Table 1 represents the 11 questions used in the checklist [18].

**Table 1:** Critical Appraisal Skills Programme (CASP) Standard Checklist

Study Evaluation Criteria
C1. Was the research question from the study focused?
C2. Was randomization of the participants towards the interventions done?
C3. In conclusion, was there accountability of the participants?
C4. Was blinding done for the following: - The participants? - The investigators? - Results analyzers?
C5. Were the study groups similar at the start of the trial?
C6. Was there a similarity in the level of care among the study groups and the participants?
C7. Was a comprehensive report on the effects of the interventions done?
C8. Was there a report on the precision of the effect of treatment or the estimate of the intervention?
C9. Were the safety and efficacies of the implantable cardioverter defibrillators identified?
C10. Was there compatibility between the population and the results?
C11. Was there a benefit in the application of implantable cardioverter defibrillators compared to those without?

Data Extraction

Two researchers independently conducted data selection and extraction autonomously (HT, MM). Where disagreements emerged, the researchers would present their basis of the argument to ensure clarity from both sides. If disagreements persisted, a third party was used to resolve the conflict.

Results

Study Selection

The initial study search identified 779 studies from PubMed and Google Scholar. These results came from using an integrated search string in the search for literature; tea OR Camellia sinensis OR green tea OR black tea OR white tea OR oolong OR CVDs OR myocardial ischemia OR myocardial infarction OR heart OR heart failure OR angina pectoris OR angina OR cerebrovascular disorders OR cerebrovascular disorder OR stroke OR cerebrovascular accident OR peripheral vascular Diseases OR peripheral vascular disease OR cardiovascular death OR cerebrovascular death. We discovered a total of 779 studies from the electronic databases, which included PubMed and Google Scholar. Using automated duplication checkers, a total of two studies were eliminated. Additional filters were used during the screening process, where 761 studies were eliminated to remain with a total of 16 studies. A further two studies were excluded from the study as they were irretrievable, thereby remaining with a total of 14 studies. An assessment by the two investigators identified two of the studies as having a poor standard of work quality. The investigators also excluded two studies for missing the standards for eligibility criteria. The study characteristics that were extracted are detailed in Table 2 [1,2,6,7,10,15,19-22].

**Table 2:** Study analysis table for the considered studies of reference TC – total cholesterol, LDL-C – Low-density lipoprotein cholesterol, HDL-C - high-density, lipoprotein cholesterol, BP – blood pressure, SBP – systole blood pressure, DBP – diastole blood pressure, CVD – cardiovascular diseases, vs – versus

Author	Aims of the Study		Intervention	Outcomes of the Study (Intervention vs. Control)
Xu et al. (2020) [1]		Assess the effect of green tea on total cholesterol, LDL, HDL, and triglyceride levels	80 to 2488.7 mg/d green tea catechin	TC: -4.66 mg/dL to -2.96 mg/dL LDL-C: -4.55 to -2.80 mg/dL HDL-C: nil
Xu et al. (2022) [2]		Effects and benefits of green tea on blood pressure	208 to 1344 mg/d green tea catechin	SBP: - 2.18 vs -1.17 mmHg DBP: -2.07 vs 1.24 mmHg
Abe et al. 2021 [6]		Outcomes of green tea focusing on diabetes, cancer sites, and cardiovascular disease	One to three cups of green tea consumption	Risk of CVDs: 36% vs 17 % reduction, SBP: -2.1 vs -1.92 mmHg DBP: -1.92 to 1.7 mmHg
Kishimoto et al. (2020) [7]		The associations between the consumption of green tea and coffee and the prevalence of coronary artery disease	Green tea consumption	CVD risk decrease: 3cups/day vs 1cup/day : 0.54 vs 0.3-0.98
Chung et al. (2020) [10]		To provide comprehensive and updated evidence on the relation between tea consumption and risks of CVD and all-cause mortality.	338 mg green tea consumption	CVD Mortality: 4% vs 2% lower risk
Yang et al. (2018) [15]		To examine the benefits of green tea	300mg green tea consumption	CVD Risk: -28% vs nil
Monnier et al. (2022) [19]		The health benefits of green tea	Three to ten cups/day green tea consumption	CVD Mortality: -28% vs 14% BP: -6.5% LDL-C: -2.6%
Abby et al. (2021) [20]		Evaluation of data on reduced CVD risks and their severity by green tea	236.6 mL green tea consumption	CVD Mortality: 0.98 vs 0.96 TC: -5.47 vs -1.67 mg/dL LDL-C: -11.02 VS -3.21 mg/dL HDL-C: -1.12 vs -0.38 mg/dL SBP: -2.80 vs -1.8 mmHg DBP: -3.06 vs – 1.27 mmHg
Teramoto et al. (2021) [21]		The beneficial health effects and possible mechanisms involved	Green tea consumption	CVD Mortality: 4% vs 2% lower risk SBP: Not indicated DBP: Not indicated
Huang et al. (2018) [22]		To examine the effects of green tea extract.	Green tea extract	LDL-C: -25.7% vs 4.8

**Figure 1:**
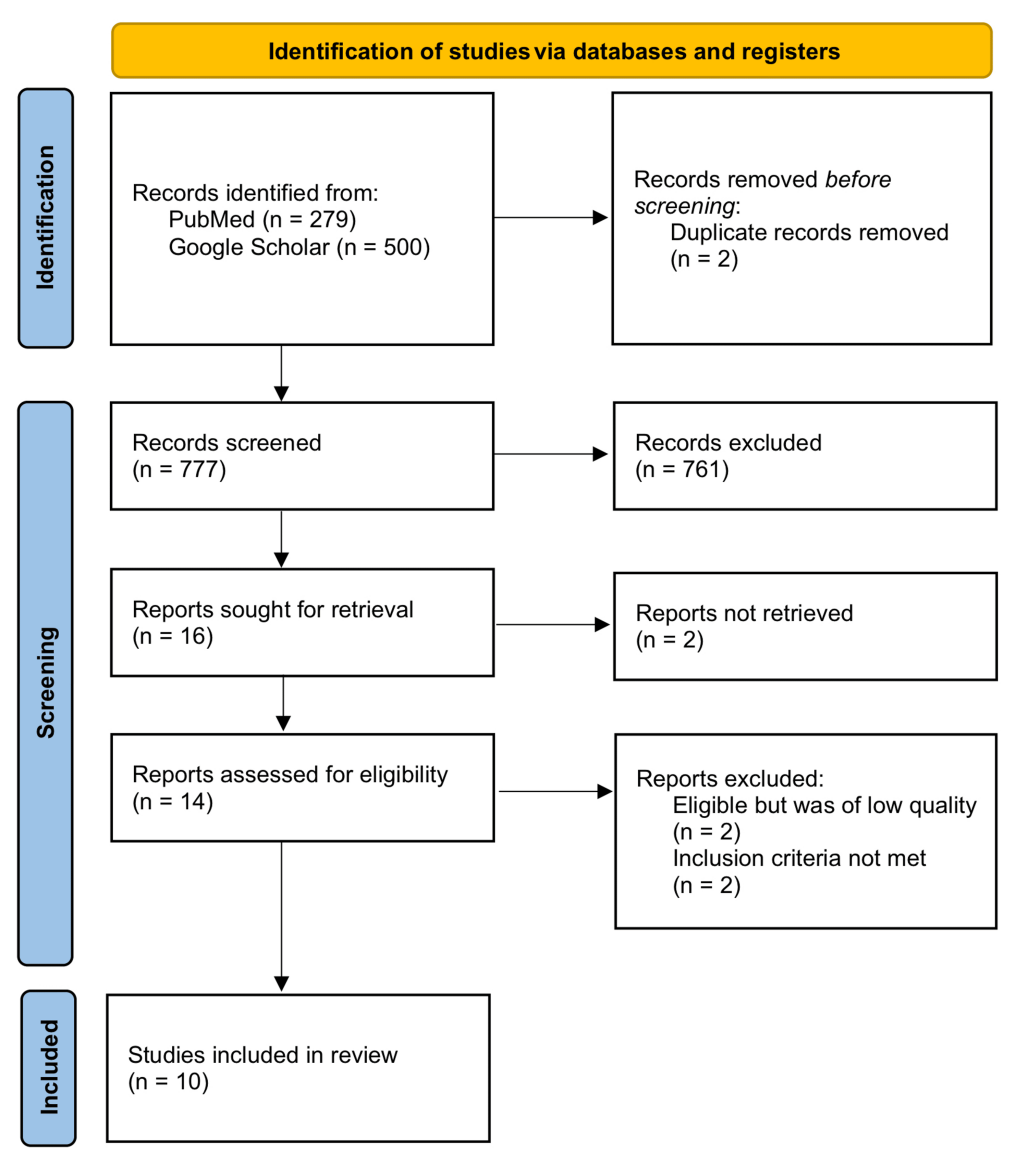
PRISMA flow diagram of the inclusion and exclusion process of the studies in the systematic review n – number of records, PRISMA – Preferred Reporting Items for Systematic Reviews and Meta-Analyses

A PRISMA flow chart was made to summarize the process for the selection of the most relevant studies that were used in the study. Summary was made according to identification, screening and the included studies. The PRISMA flow chart has a summary of the process (Figure 1).

Among the 10 studies included, five of them were systematic reviews [1,3,7,9,10], two meta-analyses [2,5], and three RCTs [4,11,18].

Quality Appraisal - CASP & RoB2

**Figure 2:**
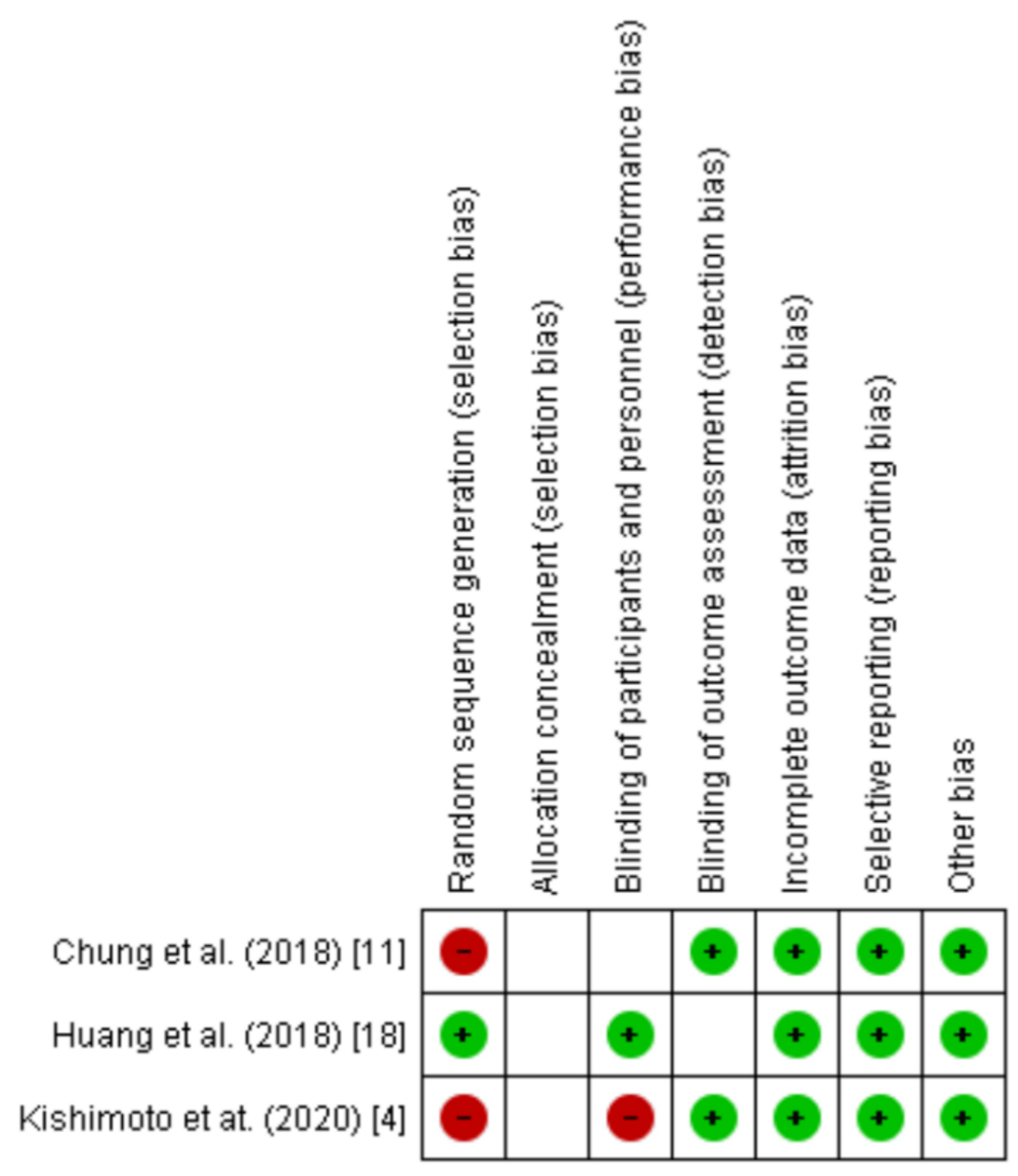
Risk of bias summary of three RCTs according to RoB2 tool

**Figure 3:**
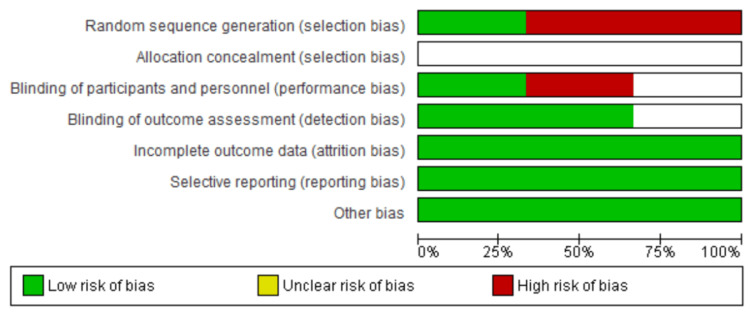
Risk of bias graph of three included RCTs

We included studies that generally demonstrated a high quality of the CASP appraisal tool. All RCTs were appraised on version 2 of the Cochrane RoB tool for RCTs (RoB2) (Figures 2, 3). Four studies [1,10,19,20] demonstrated a high-quality score (11). The rest of the studies scored between 8 and 10 on the tool and thus fell under the high-quality category. This appraisal demonstrates that the studies included in this systematic review can present reliable evidence. Table 3 shows the results of the appraisal of the included studies [1,2,6,7,10,15,19-22].

**Table 3:** CASP quality appraisal results Y – yes, N – no, CT – can’t tell

Studies	C1	C2	C3	C4	C5	C6	C7	C8	C9	C10	C11	Score
Xu et al. (2020) [1]	Y	Y	Y	Y	Y	Y	Y	Y	Y	Y	Y	11
Xu et al. (2022) [2]	Y	Y	Y	Y	CT	N	CT	Y	Y	Y	Y	8
Abe et al. (2021) [6]	Y	Y	Y	Y	Y	Y	Y	Y	N	Y	Y	10
Kishimoto et al. (2020) [7]	Y	Y	Y	Y	N	Y	Y	Y	Y	Y	Y	10
Chung et al. (2020) [10]	Y	Y	Y	Y	Y	Y	Y	Y	Y	Y	Y	11
Yang et al. (2018) [15]	Y	Y	Y	Y	Y	Y	Y	CT	Y	Y	Y	10
Monnier et al. (2022) [18]	Y	Y	Y	Y	Y	Y	Y	Y	Y	Y	Y	11
Abby et al. (2021) [19]	Y	Y	Y	Y	Y	Y	Y	Y	Y	Y	Y	11
Teramoto et al. (2021) [20]	Y	Y	Y	Y	N	N	Y	Y	Y	Y	Y	9
Huang et al. (2018) [21]	Y	Y	Y	Y	Y	CT	Y	Y	Y	Y	Y	10

Discussion

This section will discuss the effect of green tea on cardiovascular risk factors. Various systematic reviews demonstrating the use of green tea on cardiovascular outcomes have been assessed and summarized.

Risk Factors for CVD 

CVDs are conditions known to cause heart failure, coronary artery diseases, cardiomyopathy, and stroke, which emanate from the heart and blood vessels getting affected [1,2]. The conditions further increase patients' mortality, morbidity, and disability levels. The risk mechanisms involved with CVDs vary due to different diseases. An example such as coronary artery disease ranges from a stroke due to its risk effects and mechanisms [6]. Hypertension is a risk factor for both CVDs and CVD mortality. It has been identified that for each increase of 20/10 mmHg of BP, the risk of being affected by CVDs doubles. The risk applies to BP which starts from 115/75 mmHg. Ischemic heart disease has been noted to record hypertension in nearly 50% of the cases reported. Hypertension has had almost 60% of patients with stroke linked to it [1]. Many prevention therapies have been sought that deal with hypertension. They include adequate sleep, exercise, lifestyle interventions, and body weight management [7].

The presence of lipids in the body has also been factored in as a high risk for CVDs and CVD mortality [1,2]. Lipids such as high-density lipoprotein cholesterol (HDL-C) and low-density lipoprotein cholesterol (LDL-C) are examples of such lipids [10]. Exposure to high levels of LDL-C has been identified as a factor that increases the chances of CVDs among people. Lipid LDL-C reduction has been the same as HDL-C in reducing the risk of CVD mortality, strokes, and myocardial infarctions.

The studies under review noted that for every increase of 236.6 mL of green tea consumed, a reduction of about 4% was mentioned in the risk of CVD mortality [15]. For older adults aged 65 years and older, there was a reduction of 11% in the risk of CVD mortality for every 236.6 ml of green tea consumed. A study also showed an inverse relationship between the consumption of green tea and the chance of CVD events. A study showcased seven cases that showed a 2% decrease in risk events for CVD for every increase in green tea intake [19]. A study indicated four cases where the increase in consumption of green tea for every three cups was linked to a reduction of 27% of CVD events occurring in a person [20]. The study found that participants who had reported cases of hypertension, normal or high BP, or had reported other CVD risks gained many benefits from green tea. Studies further indicated that the high levels of catechins in green tea significantly influenced its ability to reduce hypertension. Studies observed participants from Taiwan who had a significant reduction in hypertension when they had a 120 mL per day intake of either green tea or oolong tea [21]. The consumption of higher levels of green tea indicated fewer hypertension cases. The population affected in this manner was reported to be more physically active and was observed to happen more in male subjects than in female subjects. The intake of green tea was observed to have the effect of lowering the risk of the prevalence of cardiometabolic risks and hypercholesterolemia [22]. This made green tea a potential for its use as a healthy lifestyle supplement.

Chemical Compositions and Pharmacokinetic of Green Tea Catechins

Green tea, black tea, and oolong tea are the most popular types of tea. Green tea contains various chemical compounds such as polyphenols, flavonoids, flavanols, and other ingredients such as amino acids, organic acids, lipids, vitamins, polysaccharides, and thiamine [23]. The main constituents of green tea are polyphenols, and the major polyphenols (catechins) are flavonoids. Green Tea has four major catechins: (-)-epicatechin (EC), (-)-epigallocatechin (EGC), (-)-epicatechin-3-gallate (ECG), and (-)-epigallocatechin-3-gallate (EGCG). The most abundant of them is EGCG, which accounts for around 59% of total catechins [24]. The next abundant is EGC (about 19%), followed by ECG (approximately 14%) and EC (approximately 6%) [25]. Catechins are the most important flavonoids, accounting for 80%-90% of flavonoids and approximately 40% of the water-soluble solids in green tea [26]. Furthermore, green tea also contains garlic acid, quercetin, kaempferol, myricetin, chlorogenic acid, and caffeine, though only half the amount is found in coffee [27].

The tea leaves are steamed or heated, rolled, and dried. Drying the leaves helps to stabilize the tea ingredients throughout storage. Polyphenols oxidize quickly after harvesting due to the enzyme polyphenol oxidase. Therefore, heating green tea leaves help inactivate the polyphenol oxidase, preventing the loss of polyphenols. Drying the leaves helps to stabilize the tea ingredients throughout the storage [12,15,27].

Oxidative stress in the body is linked to inflammation and CVD, which is caused by the harmful effects of reactive oxygen species (ROS). ROS produce chronic inflammation by inducing inflammatory cytokines, chemokines, and proinflammatory transcription factors. Green tea catechins have been shown to exhibit antioxidant effects via blocking the redox-sensitive transcription factors and pro-oxidant enzymes, scavenging ROS, and activating antioxidant enzymes [28]. ROS production suppresses deoxyribonucleic acid (DNA) and ribonucleic acid (RNA) damage and oxidation of proteins and lipids, leading to reduced cell apoptosis.

Effect of Green Tea on BP

A meta-analysis with a population study that ranged from people with morbidity to healthy individuals detailed eleven studies [2]. A significant reduction in BP after consuming the tea was noted among the population with reported hypertension and elevated BP [2,19]. The significance was only noted in the random-effects meta-analysis of green or black tea versus a placebo. The significance was not observed in the green tea versus placebo or black tea versus a placebo. A significant reduction in BP was observed in a study population of overweight or obese participants in a green tea versus a placebo in a random-effects meta-analysis [29]. A random-effects meta-analysis of black or green tea versus a placebo indicated a significant decrease in the BP of hypertensive and healthy participants. Participants with a history of vascular diseases, hypertension, and diabetes also recorded a significant decrease in the BP in a green tea versus a placebo random-effects model [21].

The green tea versus a placebo fixed effects model indicated the decrease of BP in a population of healthy participants and those in the high-risk category. The benefits were from the perspective of reduction in systole BP (SPB) and diastole BP (DBP). The process was brought about by vascular inflammation, thrombogenesis, and oxidation inhibition after drinking green tea. The improvement in endothelial functions was observed among participants with poor BP control, which reduced SPB and DBP significantly [2]. Studies also indicated more significance of the supplementation of green tea in participants who were from Western countries as compared to those who were from Asian countries. The reduction in BP was observed to be significantly higher among participants with low catechin consumption compared to those with higher intakes of the same. The difference in the composition of the green tea was its preparation methods.

Studies have indicated that caffeine increases BP in people due to its effect on increasing arterial stiffness while influencing arterial compliance [18]. However, caffeine was observed to be insignificant in the SBP and DBP among the participants of this study. Studies indicated that the composition of green tea had lower levels of caffeine when compared to catechins. This translated to having the catechins supersede their influence and effects in green tea compared to caffeine [19,28]. Studies also indicated that the supplementation of green tea among overweight and obese participants reduced BP, although it was a small reduction. The studies indicate that extracts of green tea with high catechin levels significantly reduced DBP and SBP. The consumption of green tea lowered the BP of patients who had reported hypertensive and prehypertensive ranges. Trials conducted with short-term intervals for result analysis indicated no effect of green tea on DBP and SBP [2,19]. These trials were conducted on participants who were normotensive and who had regular consumption of green tea. The insignificance was noted to be the intake of tea regularly for up to eight weeks. Longer terms in the habitual intake of green tea reported significant effects on the vasodilatory functions, ultimately leading to its influence on the reduction of BP among participants.

Effect of Green Tea on Plasma Lipids

A meta-analysis was identified with eleven studies that assessed green tea's effects on total cholesterol [2]. The demographic of the participants included a general population of people above the age of 18 years. Some participants cited hypercholesterolemia, hypertension, obesity, type-2 diabetes, and people at high risk of CVD events. The study identified a significant reduction in total cholesterol levels in obese participants with a body mass index (BMI) above 25 kg/m^2^. The study used a random-effects model of green and black tea consumption versus a placebo. A meta-analysis on green tea versus placebo or black tea versus placebo had no significant effect on total cholesterol among the participants. In a random-effects meta-analysis on the consumption of green tea versus placebo, a significant reduction in the total cholesterol was observed among obese and overweight participants and those with normal weight. Studies with smaller population sizes showed no significant effects of green tea on total cholesterol [20].

Low-density lipoprotein standardized to LDL cholesterol was identified in several studies that looked at how it was affected by green tea consumption [8]. The demographic of the participants included a general population of people above the age of 18 years. Some participants cited hypercholesterolemia, hypertension, obesity, type-2 diabetes, and people at high risk of CVD events. A significant reduction in LDL cholesterol levels was recorded in two random-effects meta-analyses among a population of obese participants with a BMI above 25 kg/m^2^ [26]. The meta-analysis was on green and black tea versus placebo and green tea consumption versus a placebo. In a study group of obese participants, overweight and those with normal weight, a significant reduction in the LDL cholesterol was observed in green tea consumption versus placebo [21]. LDL cholesterol levels were also observed to decrease among healthy participants in a green tea versus a placebo consumption random-effects meta-analysis. A study identified the reduction in LDL cholesterol levels among a population sample of diabetic and hypertensive participants in green tea consumption versus a placebo random-effects meta-analysis [21]. A Cochrane review conducted on the consumption of black tea versus green tea consumption and green tea consumption versus a placebo recorded a significant reduction in LDL cholesterol levels among a high-risk and healthy population [21]. Studies with smaller population sizes showed no significant effects of green tea on LDL cholesterol.

Several systematic reviews were identified, having assessed the effects of green tea consumption on high-density cholesterol, identified as HDL cholesterol [7]. The demographic of the participants included a general population of people above the age of 18 years. Some participants cited hypercholesterolemia, hypertension, obesity, type-2 diabetes, and people at high risk of CVD events. The studies conducted, however, did not have any significant effects on HDL cholesterol brought about by green tea consumption. Systematic reviews that assessed the effects of the consumption of green tea on triglycerides were also studied. The demographic of the participants included a general population of people above the age of 18 years. Some participants cited hypercholesterolemia, hypertension, obesity, type-2 diabetes, and people at high risk of CVD events. A significant decrease in triglyceride levels was observed among participants who had reported type 2 diabetes in a green tea consumption versus a placebo random-effects meta-analysis. Other studies did not find significant effects of green tea consumption on triglycerides.

Limitations

For the larger execution of this study, we did not face any major limitations. The methodological approach to this systematic review discovered a large number of reliable studies, which lengthened the process of inclusions and exclusion. A common limitation of these studies was the availability of useful data in the selected studies. In order to keep track of the data and the included studies, the researchers had to vet and keep track of an extensive list of studies. Our review is highly limited by the lack of consistency with study designs. By including SRs, Mas, and RCTs, we ended up looking at different population sampling, widely different methodologies, and parameters. This disparity makes it a challenge to draw generalized conclusions. Included studies do not also consider other confounding factors such as lifestyle, diet, genetics, and comorbid conditions. Failure to account for these actors ends up attributing all changes to green tea consumption.

## Conclusions

Flavonoids in green tea contribute to cardiovascular health benefits as they are enzyme modulators involved in endothelial function improvement and oxidative and inflammatory stress. CVD is influenced by dietary factors from people. Modifications in the dietary schedules are a means to regulate BP conditions among patients. Green tea consumption has a significant reduction in CVD risks. An increase in green tea consumption has a significant reduction in CVD mortality. Studies showed an inverse relationship between the consumption of green tea and the risk for CVD events. The population affected in this manner was reported to be more physically active. High levels of catechins in green tea significantly reduced hypertension. The composition of green tea was comprised of polyphenols, flavonoids, flavanols, amino acids, organic acids, lipids, vitamins, polysaccharides, and thiamine. The effects of green tea in reducing BP were observed to be very significant for both diastolic and systolic BP. Longer periods in the habitual intake of green tea reported significant effects on cardiovascular functions, ultimately influencing the reduction of BP among participants. There was a significant reduction in lipids after consuming green tea. However, no significant effect has yet been identified in reducing HDL cholesterol. The consumption of green tea has been observed to have significant positive effects on the prevalence of cardiovascular outcomes.

The effects of green tea in reducing BP were observed to be very significant for both DBP and SBP. Longer periods in the habitual intake of green tea reported significant effects on cardiovascular functions, ultimately influencing the reduction of BP among participants. There was a significant reduction in lipids after consuming green tea. However, no significant effect has yet been identified in reducing HDL cholesterol. The consumption of green tea has been observed to have significant positive effects on the prevalence of cardiovascular outcomes. More research is needed to confirm the specific impact and effective frequency of consumption. However, the positive findings from this review regarding green tea consumption for CVD health warrant clinicians to encourage incorporating green tea as part of a heart-conscious healthy diet. This systematic review does not offer an in-depth exploration of the underlying mechanisms responsible for the observed changes. To fully understand the benefits of green tea consumption on CVD, future research should focus on large-scale RCTs with long-term follow-up periods. The trials should aim to clearly understand the cause-and-effect between consuming green tea and CVD health. RCTs will allow for better control of confounding factors and more robust evidence.
