# Correction: Impact of Green Tea Consumption on the Prevalence of Cardiovascular Outcomes: A Systematic Review

**DOI:** 10.7759/cureus.c275

**Published:** 2025-08-26

**Authors:** Hadrian Hoang-Vu Tran, Mafaz Mansoor, Samia Rauf R Butt, Travis Satnarine, Pranuthi Ratna, Aditi Sarker, Adarsh Srinivas Ramesh, Carlos Munoz, Dawood Jamil, Lubna Mohammed

**Affiliations:** 1 Internal Medicine, California Institute of Behavioral Neurosciences & Psychology, Fairfield, USA; 2 General Practice, California Institute of Behavioral Neurosciences & Psychology, Fairfield, USA; 3 Pediatrics, California Institute of Behavioral Neurosciences & Psychology, Fairfield, USA; 4 Family Medicine, Kamineni Academy of Medical Sciences and Research Center (KAMSRC), Hyderabad, IND

This article has been corrected to fix the following typos in the PRISMA diagram and the first paragraph of the Results section:

- PRISMA diagram: "Reports excluded: Inclusion criteria not met (n=**3**)" has been corrected to "Reports excluded: Inclusion criteria not met (n=**2**)"
- Results: “The investigators also excluded **three** studies for missing the standards for eligibility criteria.” has been corrected to “The investigators also excluded **two** studies for missing the standards for eligibility criteria.”

